# Experiences of stigma and violence among persons affected by skin neglected tropical diseases (NTDs): longitudinal analysis from an integrated intervention in Liberia

**DOI:** 10.1186/s12939-026-02865-4

**Published:** 2026-05-06

**Authors:** India Hotopf, Shahreen Chowdhury, Hannah Berrian, Wede Seekey, Jerry Kollie, Colleen Parker, Zeela Zaizay, Emerson Rogers, Georgina V. K. Zawolo, John Solunta Smith Jr, Karsor Kollie, Tiawanlyn Godwin-Akpan, Emmanuel Zaizay, Sally Theobald, Laura Dean, Rosalind McCollum

**Affiliations:** 1 Department of International Public Health, Liverpool School of Tropical Medicine (LSTM), Liverpool, UK; 2 Pacific Institute for Research and Evaluation, University of Liberia (UL-PIRE), Monrovia, Liberia; 3 Neglected Tropical Disease Programme, Ministry of Health, Monrovia, Liberia; 4 Actions Transforming Lives, Monrovia, Liberia; 5 American Leprosy Missions, Greenville, AL USA; 6 Network for Persons Affected by NTDs in Liberia (NEPA NTDs), Monrovia, Liberia

**Keywords:** Skin Neglected tropical diseases, Stigma, Violence, Gender, Mental health, Participatory action research

## Abstract

**Background:**

Persons affected by skin neglected tropical diseases (skin NTDs) commonly face stigma and violence, which perpetuates social exclusion, mental health issues, poverty and impedes health-seeking behaviour. Thus, stigma and violence limit the attainment of World Health Organisation (WHO) 2030 roadmap progress. Evidence on stigma related to skin NTDs pertains largely to leprosy, and experiences focus on the micro level, with limited consideration of the broader meso and macro social and structural conditions underpinning experiences of stigma. This study sought to explore experiences of stigma and violence among persons affected by skin NTDs in Liberia, within the context of an integrated skin NTD programme, including the application of intersectionality theory, considering how experiences change over time and in in relation to gender and condition, and making evidence-based recommendations.

**Methods:**

This participatory action research study used participatory methods (e.g., photovoice) and worked with persons affected as co-researchers. We drew on longitudinal qualitative data (n=649 participants) from three distinct timepoints during 2019-2023 with respondents from across the health system, prioritising perspectives of persons affected. We conducted gendered thematic framework analysis, guided by a conceptual framework, drawing on the WHO violence typology, stigma forms and the social ecological model.

**Findings and discussion:**

Stigma and violence, commonly attributed to myths and misconceptions, are hindering participation and inclusion. Stigma and violence seem to have reduced, however, emotional violence and internalised stigma remain prevalent. There has been a reported decline in stigmatising attitudes held by formal health workers, but some informal providers (traditional and faith healers) continue to perpetuate harmful myths. Harmful myths and gender shape the manifestation and determinants of violence, often mirroring gender norms and converging with other forms of inequalities, with women disproportionately impacted. Leprosy was associated with the most distressing and de-humanising accounts. The relationship between skin NTDs, stigma and violence is complex and multifaceted - we propose a framework to strengthen understanding. Addressing stigma and violence is paramount in the delivery of equitable, person-centred care, with implications beyond NTD programmes. More evidence is needed to deliver tailored, gender transformative interventions that engage informal providers and community-based groups (CBGs).

## Introduction

### The relationship between skin NTDs and stigma and violence

NTDs impact 1.7 billion people globally and prevail in the most marginalised communities, perpetuating poverty and social inequities [1]. Skin NTDs, including Buruli ulcer (BU), lymphatic filariasis (LF), onchocerciasis, yaws, and leprosy, are among the top 10 causes of disability and the third most common cause of illness globally [2]. When they are not treated effectively in a timely manner, they can cause life-long disability, disfigurement, and psychosocial impacts [2, 3]. Persons affected often experience social stigma, impacting wellbeing and contributing to mental health conditions, social isolation, and exclusion from education and employment, driving generational poverty and impeding participation and inclusion [3–6].

The risk of experiencing violence is 1.5 times higher among people with disabilities, with women ten times more likely to experience sexual and gender-based violence (SGBV) as compared with men, related to their perceived inability to fulfil stereotypical gender roles [7, 8]. Experiencing violence negatively impacts mental health, health-seeking behaviour and self-management, impeding skin NTD programmes and perpetuating gender inequality [5]. The COVID-19 pandemic also exacerbated violence, namely SGBV, with the “syndemic” of violence and COVID-19 disproportionately impacting people with disabilities [9].

Hence, addressing stigma and violence against persons affected is key to achieving the WHO’s global NTD integration plan, which includes a shift towards holistic, person-centred care, considering the intersect of mental health, equity, and disability; all interlinked with violence [10–13]

### Conceptual framing of stigma and violence

**Fig. 1:**
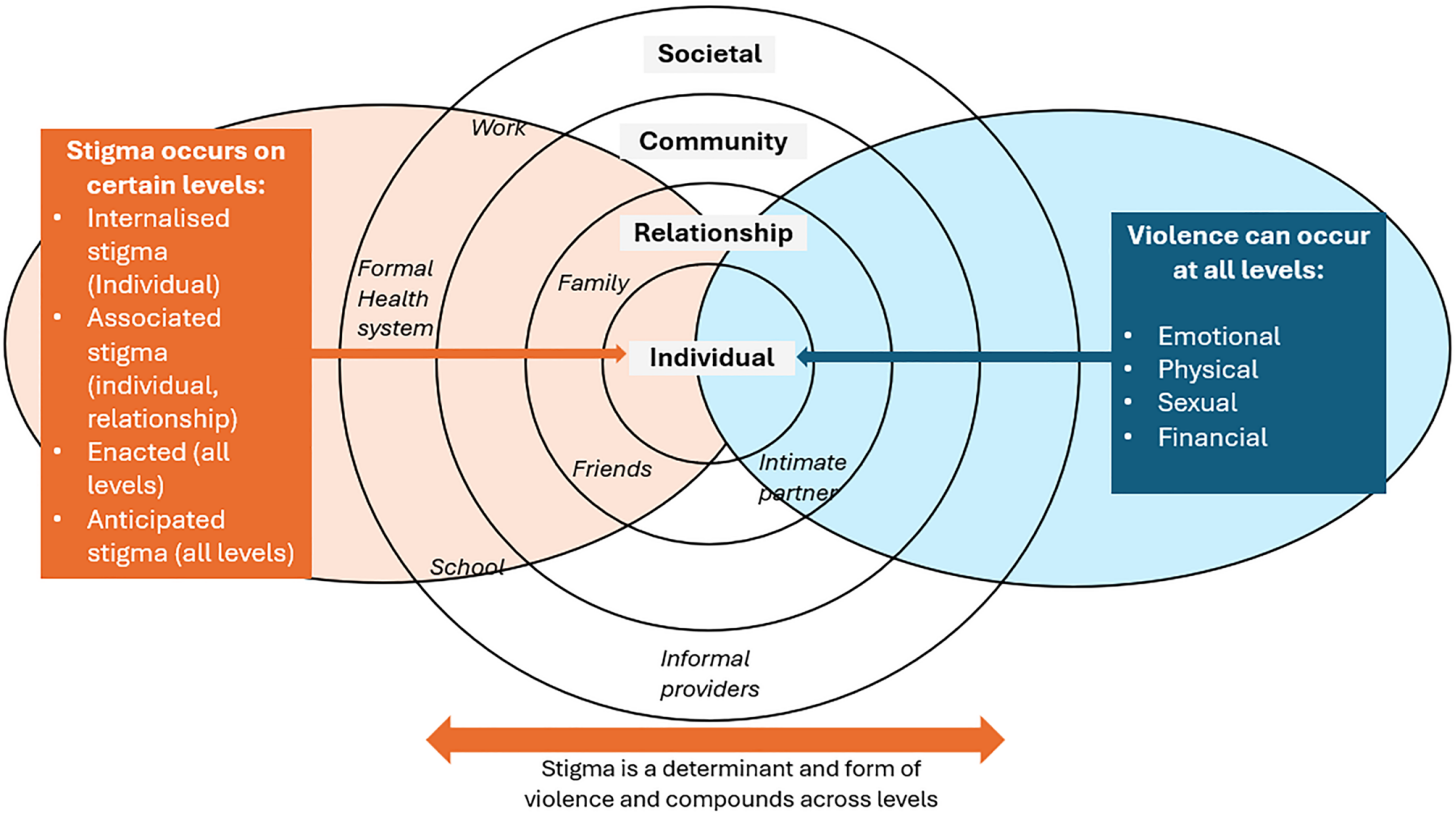
Conceptual framing of stigma and violence across levels of the social ecological model [14, 15]. Forms of emotional, physical, financial and sexual violence can occur across all levels (blue). This includes societal (schools, work, formal health system and informal providers), community, relationship (family, friends and intimate partners) and individual level. Whilst enacted and anticipated stigma (orange) can occur at all levels, associated stigma is most common at the individual and relationship level and internalised stigma occurs at the individual level. Stigma is both a determinant and form of violence and compounds across levels

The WHO typology of violence describes four forms of violence: physical (e.g., beating), sexual (e.g., rape), emotional (e.g., name calling) and deprivation or neglect (e.g., ceasing caring support) [14]. Violence can also be conceptualised based on where it occurs; the social ecological model describes four levels: individual, relationship, community and societal [15]. Stigma, defined as “an attribute that is deeply discrediting, and the stigmatised individual is one who is not accepted and not accorded the respect and regard of his peers; one who is disqualified from full social acceptances” is both a determinant and form of violence, with a mutually reinforcing relationship [16]. Through a cyclical process of labelling, stereotyping, separation, status loss, and discrimination [17], stigma normalises and justifies forms of discrimination and violence (e.g., emotional or physical violence), simultaneously perpetuating exclusion from social services (including healthcare, employment and education), “skewing their life chances” through structural violence [18, 19]. This systematic exclusion drives perceptions of stigmatised populations being ‘non-humans’ and justifies violence, whilst simultaneously impeding people’s ability to seek help [7, 8, 16, 20]. There are several forms of stigma: enacted, anticipated, internalised stigma and associated stigma [16, 21–24]. For this study, we combine the aforementioned frameworks (with some adjustments, including the addition of financial violence and the removal of neglect based on the findings), to explore different types of stigma and violence across different levels of the social ecological model (Fig. 1).

### The context of Liberia and the REDRESS intervention

Liberia is a West African country with a complex socio-political history, characterised by colonisation, and 14 years of intra-state conflict [25]. The country is experiencing an ongoing SGBV crisis, attributed to the collapse of laws, social structures and essential services experienced during the conflict, with reports indicating that up to 75% of women were raped [26, 27]. The conflict and the recent Ebola epidemic and COVID-19 pandemic placed additional strain on the health system and increased dependence on informal providers, namely traditional healers and faith healers (TH/FHs), who are typically the first port of call, due to deeply embedded spiritual beliefs [4, 28, 28–31]Due to its colonial history and numerous protracted drivers of fragility, Liberia faces high rates of poverty, health system deficiencies, aid dependency and gender inequity [4, 26, 30].

**Fig. 2:**
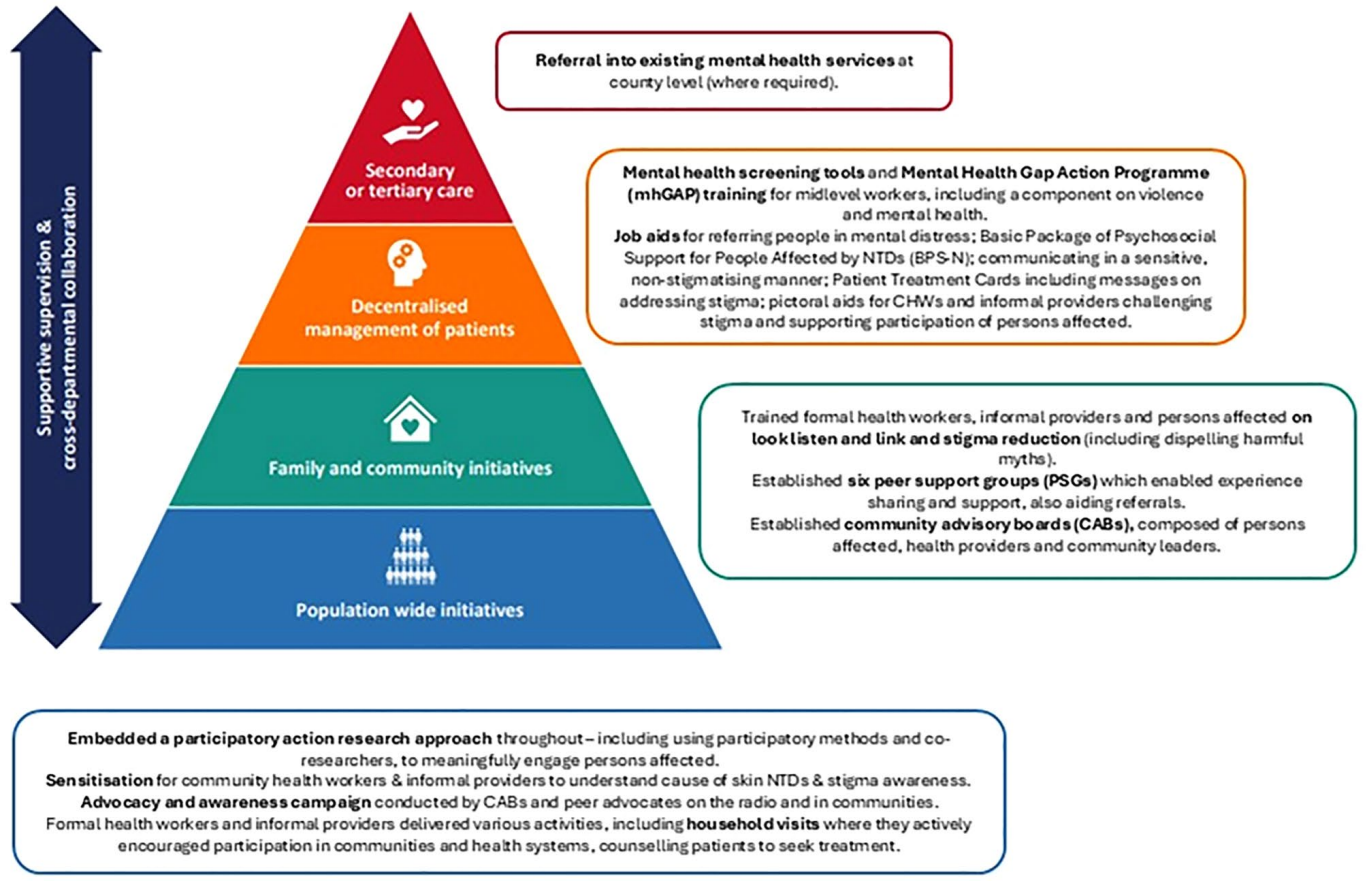
Overview of the REDRESS intervention related to stigma and violence

Liberia is endemic for multiple NTDs, including BU, LF, Yaws, Onchocerciasis and Leprosy, with high prevalence and overlaps across regions, impeding socioeconomic and gender development [32]. Despite these challenges, the country presents a rare example of a country seizing the “window of opportunity” to implement national health-system reforms post conflict [28]. It is among the first countries to implement an integrated programme for case management of skin NTDs and through the REDRESS consortium, has enhanced this through integration of mental health and stigma reduction [28]. The intervention took a holistic health system strengthening approach, engaging stakeholders from across the health system levels (including informal providers) and included various components to reduce stigma e.g., training and sensitisation (Fig. 2).

### Study rationale, aims and objectives

Whilst there is a growing pool of evidence on the relationship between skin NTDs, stigma and mental health, there is a dearth of high quality evidence exploring the inextricable link between skin NTDs and stigma and violence, especially in skin NTDs other than leprosy and in relation to social factors [3, 30, 33–37]. To our knowledge, no published studies have utilized longitudinal participatory methods to explore the phenomenon with a focus on lived experiences of persons affected by skin NTDs, who’s experiences are often neglected or overlooked [34]. Evidence is urgently needed to enable the design and implementation of evidence-based, tailored stigma reduction interventions to support the delivery of equitable, person-centred care as outlined within the WHO [38] Essential Care Package to address mental health and stigma for persons with NTDs (ECP), and achieve the WHO 2030 Roadmap targets [3].

Hence, this study sought to explore experiences of stigma and violence among persons affected by skin NTDs in Liberia over the REDRESS intervention time period, through the following objectives: i) explore how experiences of stigma and violence changed over time ii) elucidate how gender and condition intersect with other axes of inequity to shape experiences of stigma and violence and iii) consider the determinants and implications and make evidence-based recommendations for addressing stigma and violence, strengthening the delivery of equitable, person-centred care.

## Methods

### Study setting and design

This paper primarily draws on longitudinal data from the REDRESS programme – a participatory action research study, which co-designed and evaluated an intervention for integrated skin NTD care [30]. According to its participatory principles, REDRESS meaningfully engaged person with lived experience throughout the research cycle as paid co-researchers [39]. Co-researchers contributed to data collection, analysis and intervention design, through attending and presenting at participatory workshops. Additionally, co-researchers acted asmembers of community advisory boards, which included other persons with lived experience [40].

The study was embedded in the naturalistic paradigm and used participatory qualitative methods to “investigate the meaning of social phenomena as experienced by the people themselves” [39, 41]. An intersectional approach was applied throughout the research cycle, including designing tools that explicitly explored intersecting axes of inequity, sampling participants with maximum variation for age, gender, disability and geographical location, as well as recruiting participants from across the health system [42]. Data collectors (including co-researchers) were also trained on power and positionality and kept reflexive diaries throughout. During the longitudinal gendered analysis, there was also a conscious effort to highlight how multiple intersecting inequalities shaped experiences of skin NTDs, stigma and violence, using gender as an entry point. As well as to consider the drivers of inequality, by considering how the dimensions of social identity interact with social processes and structural factors to drive discriminatory beliefs, which contribute to the varied experiences of stigma and violence by persons affected by NTDs [43].

Additionally, we drew on data from LD’s PhD linked to REDRESS and an evaluation funded by The Coalition for Operational Research on Neglected Tropical Diseases (COR-NTD) (Table 1). The COR-NTD project evaluated the existing case detection and referral approaches in Liberia from 2019 to 2021, which was used to develop and test an optimal model for integrated community-based case detection, referral, and management [44]. This included data from the Bong County, hence its inclusion.

**Table 1:** Summary of data included in study, including the total number of participants (*n* = 649) and total number of data points (*n* = 208). Unless otherwise indicated, FGDs included 6–8 people, therefore 7 has been used as the average number per group

Timepoint	Dataset	Method	Participant type	County	Number of participants		
					Women	Men	Mixed
Formative (2019–2021)	COR-NTD Evaluation	Focus group discussion (FGD)	Community health workers (CHWs)	Bong	-	-	28 (4 groups)
			Peer advocates	Bong	-	-	7 (1 group)
	LD PhD	In-depth interview (IDI)	Persons affected and family members of persons affected	All	19	20	-
	LD PhD	Illness narrative (ILN)	Person affected	All	30	24	-
	COR-NTD Evaluation	Key informant interview (KII)	Community health service supervisor (CHSS)	Bong	-	-	4
	REDRESS	KII	Skin NTD professionals	Montserrado	3	3	-
	COR-NTD Evaluation	Reflexive sessions	Research team	Unassigned	-	-	7 (1 group)
			NTD Program	Unassigned	-	-	21 (3 groups)
		COR-NTD report	CHSS	N/A	-	-	-
	REDRESS	Vignette activity	Patient advocates	Bong	1	2	-
Baseline (2022)	REDRESS	Body mapping	Community member	Grand Gedeh	7 (1 group)	7 (1 group)	-
		Social mapping FGD	Patient advocates	Grand Gedeh	7 (1 group)	-	-
			Patient advocates	Grand Gedeh	-	7 (1 group)	-
			Patient advocates	Lofa	7 (1 group)	7 (1 group)	-
			Patient advocates	Margibi	7 (1 group)	7 (1 group)	-
		FGD	Community member	Grand Gedeh	7 (1 group)	7 (1 group)	-
			Traditional healer	Grand Gedeh/Margibi	-	-	14 (2 groups)
			Faith healer	Margibi	-	-	7 (1 group)
			CAB members	Grand Gedeh/Lofa/Margibi	-	-	21 (3 groups)
			Community health actor/community health volunteer (CHA/CHV)	Margibi/Lofa	-	-	14 (2 groups)
			Person affected	Margibi	-	-	7 (1 group)
		IDI	Person affected	Grand Gedeh	2	1	-
			Person affected	Lofa	-	2	-
			Person affected	Margibi	2	2	-
		Photovoice	Persons affected	Grand Gedeh	-	-	7 (1 group)
		Reflective diary FGD	Faith healer	Grand Gedeh	-	7 (1 group)	-
			Officer in charge (OIC)/CHSS/CHA	Grand Gedeh	-	14 (2 groups)	-
			Persons affected	Grand Gedeh	7 (1 group)	-	-
			OIC/CHSS/CHA	Margibi	14 (2 groups)	28 (4 groups)	-
Endline (2023)	REDRESS	FGD	Faith healer	Grand Gedeh/Lofa/Margibi	-	-	21 (3 groups)
			Traditional healer	Grand Gedeh/Lofa/Margibi	-	-	21 (3 groups)
			PSG person affected	Grand Gedeh/Margibi	-	-	14 (2 groups)
			Person affected	Lofa	-	-	7 (1 group)
			CHA/CHP	Grand Gedeh/Lofa/Margibi	-	-	42 (6 groups)
			Facility health worker (HW)	Grand Gedeh/Lofa/Margibi	-	-	21 (3 groups)
			District health management teams (DHMT)	Lofa/Margibi	-	-	14 (2 groups)
		Reflexive diary FGD	Persons affected, traditional healers and faith healers	Grand Gedeh/Lofa/Margibi	-	-	18 (3 groups)
			OIC/CHSS/CHA	Grand Gedeh/Lofa/Margibi	-	-	21 (3 groups)
		FGD	Persons affected	Grand Gedeh	14 (2 groups)	7 (1 group)	-
			Persons affected	Lofa	-	21 (3 groups)	-
			Persons affected	Margibi	14 (2 groups)	7 (1 group)	-
		KII	MoH and partners	Unassigned	-	10	-
			NTD focal person (NTDFP)	Grand Gedeh/Lofa/Margibi	-	-	3
			Mental health focal person (MHFP)	Grand Gedeh/Lofa/Margibi	2	1	-
			Community health focal person (CHFP)	Grand Gedeh/Lofa/Margibi	1	1	1
				Total participants	144	185	320

Data collection was conducted across five counties: Montserrado, Bong, Nimba, Lofa and Grand Gedeh. Counties were purposively selected in collaboration with the Liberian MoH based on whether they were 1) endemic for skin NTDs 2) piloting the integrated case management programme and 3) geographic and socio-cultural diversity. Nimba county is home to Ganta Leprosy Rehabilitation Centre which previously served as a leprosy colony and continues to provide specialist care [45]. The REDRESS intervention was implemented in Margibi, Lofa and Grand Gedeh between 2022 and 2023.

### Data collection

**Fig. 3:**
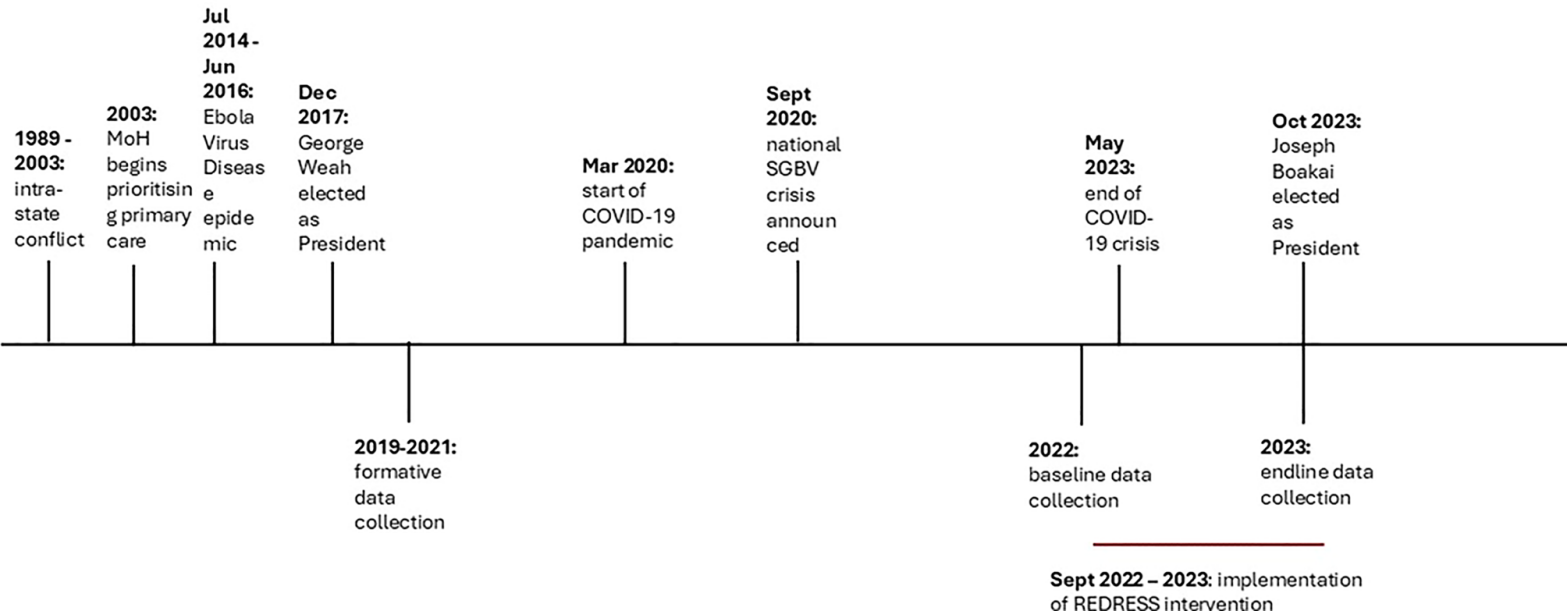
Timeline of key events in Liberia, in relation to skin NTDs and stigma/violence

We draw on longitudinal data from three distinct timepoints, each with unique contextual considerations: Formative (2019–2021), baseline (2022) and endline (2023) (Fig. 3).

REDRESS used a combination of qualitative and participatory methods to engage a range of different stakeholders, including persons affected, formal health workers and informal providers and community members (Table 1). For all three datasets, participants were purposively selected based on disease condition, professional experience and geographical location, with maximum variation for gender, county, job role and professional experience. Within the COR-NTD evaluation, participants were also purposively sampled based on their involvement in the optimal model being evaluated. Participants were identified following discussion with County level NTD staff, who facilitated introductions. All audio files were transcribed verbatim and stored securely, with 10% randomly quality checked.

Broadly, data collection tools explored participation and inclusion of persons affected within their communities, before and after the intervention, including the impact on mental wellbeing and experiences of stigma and violence across the health system levels. Most data was collected by Liberian research fellows and co-researchers, with support from the LSTM team who also contributed to some data collection (IH, SC, HB, WS, JK, CP, ZZ, ER, GZ, JSS, KK, TGK, EZ, ST, LD, RM). Please note that some of the data has been published elsewhere [4, 28, 46].

### Data analysis

All qualitative data was analysed using the thematic framework approach to help classify and organise the data according to the main themes, categories and codes emerging from the data [47]. Researchers familiarised themselves with the data and formulated a deductive framework (based on the topic guide) initially, with inductive themes iteratively incorporated throughout the coding process. For baseline data, co-researchers reviewed transcripts together, contributing to development of the coding framework. The coding framework was then applied to data, supported by Nvivo 12, to help manage data as part of analysis. After coding the data, charts were developed which considered gender, type of condition, location of participants, which were exported from NVivo into an Excel file. Next, descriptive summaries were produced, including analysis of these axes of inequity [48].

For this paper, all original qualitative data from each timepoint (*n* = 249 Excel framework matrices and one COR-NTD report) was reviewed by the lead researcher (IH).All data related to participation and inclusion, stigma, violence and psychosocial wellbeing was extracted and re-coded in one combined Excel file, according to the conceptual framework (e.g., column of relevant data extracted for experiences of stigma and violence within work place) (Fig. 1). Next, IH summarised the findings in a Word document, according to the conceptual framework and selected illustrative quotes. Analysis took an intersectional approach, using gender as your entry point and drawing on the socio-ecological model to consider meso and macro social and structural processes driving inequity

### Trustworthiness

To promote data quality, 10% of transcripts were randomly selected and quality checked, and all data collectors received training and support in qualitative research. Trustworthiness was assured through triangulating different participatory methods, involving a range of stakeholders, including persons affected, in analysis and fair dealing, wherein a range of different perspectives were included through ensuring maximum variation for gender, age, disease condition etc. To aid reflexive practice and enhance credibility, researchers from the UK and Liberia engaged in regular meetings to debrief and reflect on their experiences as a team. During the analysis and write up of this paper, the lead researcher (IH), who is a white European woman, practiced researcher reflexivity [43]. This included keeping a reflexive diary and engaging in critical discussions with the wider research team, including Liberian colleagues. Please see the ‘strengths and limitations’ section for reflections.

### Ethics

Ethical approval was obtained from the LSTM Research Ethic Committee, United Kingdom (20–040) and the UL-PIRE Institutional Review Board (20–09-233) in March and April 2020, respectively. The COR-NTD study was approved by the UL-PIRE Institutional Review Board (NTDSC 167D). For LD’s PhD, ethical approval was granted from LSTM (16–070) and the UL-PIRE Institutional Review Board (17–02-024). Our research was conducted in accordance with the Declaration of Helsinki.

All participants were adults who provided informed consent, with the voluntary nature of the study emphasised. Data collection was conducted in private locations, with all identifiable information removed and unique codes assigned, to ensure confidentiality. Data was stored securely, with access restricted to core team members. Given the sensitive nature of the topic, prior to data collection, support services for referrals were mapped and all qualitative data collectors were trained in identifying and responding to signs of distress, in line with the REDRESS safeguarding plan. Where required, respondents were provided with refreshments and reimbursed for travel costs. During analysis, care was taken to ensure that lived experiences were represented with dignity and respect, including avoiding sensationalism, when selecting quotations to include.

## Results

To foreground our findings, we include a brief reflection on the social processes and structural factors which strengthen or subvert inequalities experienced by persons affected. Liberia’s context includes post-conflict, high levels of SGBV, poverty, and a weakened health system, with low educational levels and strongly held supernatural beliefs [4, 25, 26, 30]. Together these influence various discriminatory beliefs held by others, including individual, family, community and both formal and informal providers (such as sexism, ableism, phobia of perceived supernatural illness), contributing to a loss of power and the experience of stigma and violence described by persons affected by NTDs in our study [30]. Below, we present experiences of stigma and violence, including determinants and impacts, across the four levels (societal, community, relationship and individual) (Fig. 1) and three timepoints. Experiences of violence are explored in relation to 1) time 2) gender and other social dimensions and 3) disease condition, in relation to other intersecting inequities and social dimensions.

### How did experiences of stigma and violence change over time?

In this section, we explore how reported experiences of stigma and violence, including determinants and impacts, manifest and change through time across the four levels, which intersect and compound with one another. Within this, we explore different forms of stigma and violence. Note that the intent is not to quantify the prevalence of violence, or establish a causal relationship, but rather explore how experiences may have shifted over the formative (2019–2021), baseline (2022) and endline (2023) intervention timepoints.

### Societal

Overall, we found that participants’ accounts of stigma in schools, within the formal health system and the workplace reduced from 2019 to 2023, especially in schools. However, it continues within the informal health sector.

School-based violence, resulting in dropouts, was reported by several women during formative data collection. This was largely attributed to girls feeling unable to wear skirts and their experience of emotional violence, such as insults, which originated from the communities fear of perceived supernatural origin of the condition (belief that she was a witch). This discriminatory belief intersects with the gender and age of those affected (female, youth), within a setting where sexism and greater respect for adults compared with youth contribute to a low level of power, contributing to their experience of emotional violence. As a result, this perpetuates gender disparities in educational attainment and in one case, resulted in suicidal ideation. A couple of respondents also described drop-outs due to socioeconomic constraints, including a young woman who was forced to engage in sex work to afford housing, and a man affected whose wife reportedly abandoned him and withdrew their children from education, illustrating intersections with socioeconomic constraints and between the societal and relational levels (Fig. 1). However, there were no accounts reported at baseline and endline, with one young woman reportedly able to rejoin school, following the REDRESS intervention.

*When I see my friend, them going to school I can feel bad […] Some people say that [it was] witch and I myself [too] I believe it that was witch […] when we came here the people tell us say oh that [is] not witch […] They start encouraging me then I myself started feeling fine. They said we will treat you then you will go [back] to school […] I used to do everything inside. I just feel say I am coming die. Sometimes when I sit down for long my ma [mother], when she looks at me, she can be crying. [Ok, so you just used to be thinking about dying? Interviewer speaking] yes. (Female affected by BU, ILN, formative phase)*

Reports of work-based violence were slightly more common and primarily linked to physical condition, with some accounts of emotional violence within formative and endline data (including colleagues refusing to work alongside persons affected and name calling/gossiping). Also at endline, a health worker reported driving a woman whom he purchased rice from out of their community with his colleagues, due to transmission misconceptions Consequently, some persons affected described taking up informal employment, such as sex work and begging or selling on the street, illustrating the intersection with livelihoods.

Persons affected by NTDs in Liberia experience complex journeys which are shaped by belief systems (such as supernatural beliefs about NTD causation), (loss of) confidence in the provider (for example due to drug stock outs within formal health system), (lack of) acceptability of the provider (for example providing caring/harsh care), geography and poverty (for example being unable to afford transportation to reach formal health system) [30]. Moreover, several key informants asserted a lack of recognition for the importance of mental health within Liberian society as a whole, demonstrated by mental health services only being introduced in the past 10 years and a lack of specialists. Additionally, some highlighted stigmatisation towards mental health specialists from other health workers, including referring to them as “crazy people doctors”.

Some people affected described experiencing violence from formal health workers during formative and baseline phases, due to myths and misconceptions, impeding health-seeking behaviour and acceptance, but this reportedly reduced over time. Manifestations of violence from health workers typically included emotional violence (e.g., name calling - e.g., “the leprosy patient”, “rotten man” and “aggressive” nurses), financial violence (e.g., overcharging), and inadequate confidentiality. Less commonly, narratives included patients being denied healthcare or physical touch due to misconceptions and discriminatory views. In a mixed gender FGD at baseline, mockery, apathetic communication and financial violence was commonly described, with one person affected concluding they would “rather die” than seek formal healthcare, illustrating the convergence of structural violence.

However, at endline, many health workers and persons affected described a positive shift, characterised by relationship building and long-term follow-up, with common sentiments of recognising persons affected as human beings who can be cured, and expressing regret at their prior behaviour. These changes indicate how taking part in training and recognising the health system’s ability to provide effective treatment, resulted in a change in previously held discriminatory beliefs and attitudes towards persons affected by NTDs, including that skin NTDs are incurable. There were no accounts of ongoing stigma and violence from health workers described by persons affected at endline.

*We never knew that there was treatment for them, and we used to keep them aside. We don’t want to go closer to them and they feel that they are not human. But from the training we begin to know that there is treatment for those people, and they are human like us. No need to discriminate them from us […] So, people are running to the hospital now (Health worker, reflective diary FGD, Grand Gedeh, endline phase)*

However, several informal providers asserted ongoing violence from formal health workers, including health workers reportedly refusing to refer informal cases until they have exploited people of money and delayed them so much that they will likely die. One FH asserted that persons affected are “scared” to visit hospitals, due to financial violence, and a few reported persons are treated with a lack of care, causing them to leave.

Informal providers play a key role in care for persons affected by NTDs, with persons affected typically experiencing complex care seeking journeys in search of a cure, including both formal and informal providers. During the formative phase, we elucidated the typical role of TH/FHs [49], which we will briefly provide for context. Typically, FHs wait for visions about clients a couple of days prior to initiating treatment, which varies depending on the type of church. Some might use prayer or fasting to treat skin NTDs, whilst those in the ‘Aladura church’ might perform practices such as chaining people up. THs might ‘play gamble’ (ask higher powers) to ascertain whether patients will be successfully treated, whilst herbalists apply ointments or leaves, or might necessitate that patients provide rice, white cloth, oil or a goat for sacrifice, especially if the cause is deemed witchcraft. Other THs diagnose patients through dreams; if the dreams indicate a biomedical cause, they reportedly refer patients to formal providers. On some occasions the approach to treatment entails stigmatising and isolating patients, due to beliefs that NTDs and mental health illnesses are caused by witchcraft. Often family members or relatives are considered responsible for witchcraft.

Violence from informal providers was seemingly more prevalent than formal health workers, with many accounts during the formative phase, declining over time. Respondents commonly emphasised TH/FHs role in perpetuating harmful myths (e.g., implicating friends or family in bewitchment), driving stigma across community and relational levels (Fig. 1), as well as providing costly, ineffective and sometimes harmful treatments (e.g., pouring boiling water on sores). Some THs/FHs also demonstrated discriminatory views during interview (e.g., describing persons affected as “crazy persons”) and reported keeping people against their will.

*I [don’t] believe in country doctor […] [when] you go to them, they will tell you whole lot to spoil your mind […]They will sometimes tell you that your mother, sister or grandmother is the cause of your illness. (Female affected by leprosy, ILN, formative phase)*

Some persons affected felt unable to reject traditional care, due to the strong community beliefs, and some asserted that TH/FHs wanted to “destroy” them through draining money and refusing formal referrals (described more fully in McCollum et al. [30].

At endline, many informants, primarily formal health workers, continued to warn of patients facing violence when accessing informal care having previously engaged with the formal health system (and vice versa). They emphasised THs/FHs as critical sources of stigma and misinformation. This is particularly problematic, given TH/FHs influence and community trust placed in them, illustrated by one informant describing TH/FHs as “demigods” or cultural representativest. However, over time only a couple of persons affected described that TH/FHs continue to charge high treatment costs, including demanding chickens, in exchange for ineffective treatment. Indicating how persons affected experience potential financial exploitation from informal providers, due to their desire for a cure, persistent supernatural beliefs and perception that the health system is not able to provide effective care. Many key informants warned against persistent challenges with discriminatory views and myths, demonstrated by some THs/FHs. This illustrates how stigma, cultural authority, weak health system integration and socioeconomic vulnerability converge across multiple levels (Fig. 1) to limit the autonomy of persons affected by skin NTDs.

*I think they play a key role because neglected tropical diseases are conditions that are heavily tied around stigma, and in the communities, especially around Liberia, you get to know that traditional healers form part of the society or form part of the culture that either breaks the barrier or increase the stigmatisation. (Male national partner, KII, endline phase)*

### Community

At endline, many people, primarily PSG members, described declines in violence and increasing support from communities, which they linked to improved physical condition, myth busting to counter supernatural beliefs, and patient advocate roles, reportedly humanising persons affected. Whilst accounts of physical violence in particular, declined, emotional violence remained more pervasive at endline, with limited accounts of SGBV.

CAB community members commonly described how involvement in groups changed their own attitudes and empowered them to reduce community stigma, for example through conducting awareness sessions in community markets. Such sessions reduced stigma, through addressing supernatural beliefs and misinformation, educating communities and persons affected on effective health care and humanising persons affected by skin NTDs.

*I feel that is my responsibility as community person to be able to give back to the community more especially to identify the people who are being stigmatised, who found themselves in the state that “yes, I am nobody who can identify with me” […] itsmy responsibility to be able to give back to the community to identify with people and say “yes, you can still be part of society (Mixed gender CAB members, Margibi, FGD, baseline phase)*

However, some persons affected, and informants described persistent emotional violence from communities at endline, including a person who reportedly faced social stigma within their PSG. Community-level physical violence (e.g., attempted drownings, beatings, forcible displacement from communities), largely linked to witchcraft allegations, was described by several persons affected and many informants at the formative phase.

*When I was accused as witchcraft in 1996, I received heavy beating […] that hit my nut seed [scrotum] it caused me … I was out of my sex. In 2003, they took out the thing. I just like that. So, she [wife] too, she can’t be like that. So, she went back to her old husband (Male affected by leprosy, Montserrado, formative phase)*

Also regarding physical violence, there were a few distressing secondary accounts at baseline, including two men who were imprisoned in the bush; one was treated like a “dog” until he died, whilst another described being covered in flies with everyone “looking at me”. These accounts illustrate how in a setting of rurality, low education, high poverty, lack of confidence in the formal health system, with strongly held supernatural beliefs, collective dynamics justify and perpetuate discriminatory beliefs which dehumanise persons affected, contributing to physical violence and neglect.

*Sometimes they build this palm thatch something, the build it in the bush and they put the person there. It happened to one man they called [name], he left there until he grew all over and he died behind our town, yes. Nobody, going to him, they can just throw his food, or they set it down and they be pushing it with the long stick to go to him in that fence.*

*They didn’t carry him to hospital, and they never wanted to go around him. They said he was so wicked and the name that was on him, they said he was part of this hard man thing (ritualist); so they; looked at him and his own was worst. So, people used to go and chunk him with fire chunk and all.*

*They fed him like dog! When they eat their food, the balance one they just put it in pan. He had special pan; they will just go dump it inside and push it in the fence. Ants getting on him and all kinds of things and he will be crying over night but nobody to pay attention to him; his own of judgement was done right here, yes. (Female persons affected by unassigned NTD, Margibi, IDI, baseline phase)*

A few community members also emphasised that persons affected are often “used as a torchlight for witchcraft activities”. Whilst there were no accounts at endline, some formal health workers emphasised ongoing issues with beatings and bush exiles, due to misconceptions (witchcraft, incurable and hereditary), perpetuating fear and informal health-seeking behaviour, illustrating the cross-level influence across the societal, community and individual levels (Fig. 1). There were limited accounts of SGBV, except for some key informants asserting rape and harassment was common and one woman affected by an unassigned skin NTD describing experiences of molestation during the baseline stage.

Emotional violence, also driven by harmful myths, was common at formative and baseline and typically involved exclusion socially (e.g., from meetings, groups and church) and physically (e.g., from communal pumps), as well as dehumanising insults. During the formative phase, one man with lived experience recalled an older man with severe leprosy being forced to live in a tent upon discharge. Children taunted him, echoing his own experience. The man emphasised his desire to be buried in Ganta (the town where the national leprosy hospital is located), where he felt treated as a human, demonstrating the contextual dependency of how the experience of stigma and violence changes based on location (or over time) [43]. Since persons living in the community surrounding Ganta have greater familiarity and experience with leprosy, this person did not experience the same level of discrimination whilst living there, as compared with a community in a different location.

Likewise, formal health workers commonly emphasised the detriment of harmful myths and the importance of awareness and counselling to increase education, and combat discriminatory beliefs about the cause of NTDs, to help overcome persistent community stigma, manifesting as emotional violence. Participants commonly asserted that emotional violence drove poor mental health, self-stigma and reduced participation in health systems and society as a whole, illustrating the cumulative reinforcement across levels (Fig. 1).

### Relationship

Reports of familial violence declined at endline, wherein there were only some accounts of violence and respondents described increased acceptance, linked to their improved physical condition. Familial violence (including from in-laws) was commonly reported at formative and baseline. Accounts were characterised by physical and or social exclusion (e.g., from meals, sharing household objects), halted support and resulted in abandonment, primarily due to witchcraft allegations, transmission fears and perceived inability to contribute to households and communities, illustrating intersections with norms of ableism and the importance of economic productivity, within a setting of high poverty. This included several accounts of physical violence against women at the formative stage, as well as a man who was physically chained up by his family to prevent suicide. A married couple affected by leprosy also recalled their families confiscating their food, farmland and babies.

*Your people can’t highly accept you the way they supposed to accept you. They can be afraid of you now. Even my, my children, many of them they are outside there […] when you want to eat with them some of them say ‘you are sick’ […]so it make me I left them, then the family that here now, they are my family because; you are sick, I am sick, when you and I are together, I will not feel hurt. (Male affected by leprosy, Wuo Town (ex-leprosy colony) Nimba, ILN, formative phase)*

There were several accounts of associated stigma at the formative stage, primarily in terms of emotional and physical violence. This included families and partners of people affected by skin NTDs being excluded from communities (e.g. socially or from accessing materials for bathing), being denied food and water, insults and one woman’s mother being beaten and sent to the bush. This was largely attributed to families refusal adhere to community norms and abandon persons affected, as well as transmission fears. There were no accounts at baseline and just one at endline. In fact, at baseline, a couple of women emphasised experiencing community support and appreciation for caring for their blind husbands affected by skin NTDs.

One woman affected by leprosy asserted that post-war, leprosy patients were refused treatment and killed by families who refuse children with disabilities, demonstrating intersections with societal level forces, including Liberia’s post-conflict setting. Respondents frequently described the detrimental, dehumanising impact of their own children fearing them and being unable to lie or eat with their family. This caused severe mental health impacts, including suicidal ideation, self-stigma, reintegration fears and challenges meeting basic needs, in some cases.

*So, I am thinking, before going there, even to go back to it, I don’t know how really I will live among that family […] When I think on it yesterday, I cry because if this sickness get on you, you neglected […] Almost my one year here, none of my family reach to me. I thinking on that, how will I go and live with them … .how will I go and live among the people. […] because of condition now, you just look that kind of way, they neglect you. That kind of bad feelings can come to you. That how you can just say, but let me just harm myself let everything finish because I don’t want to live alone […] That, that kind of feeling I thinking on, that what was in my heart yesterday. (Female affected by leprosy, Ganta rehab, ILN, formative phase)*

Emotional violence from friends was relatively common at formative, typically characterised by peers ceasing contact, exclusion from social events and insults (e.g., “rotten”). This drove self-stigma among persons affected and contributed to mental health issues, illustrating the intersection between the relationship and individual level. There were no accounts of physical violence from friends. This changed over time, with just one account of emotional violence from friends at both baseline and endline. Most respondents commonly described reconnecting with friends, due to improved physical condition and PSG association, with just one account of persistent violence.

*The thing I like about the peer support group here is they can talk to people too good […]. Since the group […]My friends are coming near to me. And when they go, they talk to me to good. They hold me good […] Before my friends them come move from behind me again. (Mixed gender PSG members, Margibi, FGD, endline phase)*

Spousal abandonment was commonly reported at the formative stage, declining with time, with just one account at both baseline and endline. Abandonment was commonly attributed to pressure from families, in-laws and communities around the perceived inability to perform gendered roles.

*She and myself had the children, if she can leave me say, you are sick they say you have leprosy […] You see she died and she and myself had children but because of sickness she too say the family member told her say ‘the man who is sick, you keep yourself with the man what it will profit you? Nothing you will get from the man’. So, she too she go […] even when I got this sickness, I even decided to kill myself, but that God just make way (Male affected by leprosy, Wuo Town (ex-leprosy colony) Nimba, ILN, formative phase)*

However, at the formative stage, many persons affected highlighted their families, including spouses, parents, siblings and children, as the most critical source of support. Often, support from a sibling or parent was the sole support available and occurred within the context of active stigma and exclusion from other family members. Support included childcare, cooking and household chores (e.g., collecting water), contributing to farm work or taking up additional employment. At endline, persons affected described a shift towards support and acceptance from wider family members, with health workers also emphasising the importance of garnering family support during the treatment journey.

### Individual

Internalised stigma, driven by feeling like a burden, internalising myths (e.g., witchcraft and transmission) and insults, their physical condition (e.g., odours and leaks) and being unable to wear certain clothes (e.g., school skirts) was frequently reported during the formative phase, with several accounts at baseline. Self-stigma commonly resulted in shame, self-isolation (e.g., due to fear of infecting others), severe mental health impacts (e.g., anxiety, suicidal ideation and suicide in one case) and negative self-perception of being “useless” or a “baby”, due to their loss of independence and self-sufficiency. This ultimately resulted in self-neglect and isolation, even in the face of encouragement from those around them. For instance, persons reported sequestering themselves at home or even the bush, impeding health-seeking behaviour and social participation, illustrating the compounding impact across multiple levels (Fig. 1). Health workers commonly emphasised the role of themselves and the community in helping people realise “they are human too” and increasing participation in health systems and communities, to combat stigma.

*She started isolating herself. In fact, she started telling the children say I don’t want you people to come closer to me because I don’t want you to get this sickness that I get […] It’s not that they did not want to care for her, but she actually put herself aside because she didn’t want the other family members to get it the sickness […] her sister was the best buddy to her, so all her secret, the sister had to know it. […] Even her sister, she can’t allow her own sister to come around her] (Female affected by leprosy, Nimba, ILN, formative phase – translator)*

At endline, many persons affected, primarily PSG members, described improvements in their self-perception (e.g., learning “how to love ourselves”) and recognising their humanity. This was echoed by many formal health workers and reportedly increased participation of persons affected. However, several persons affected and many health workers warned against persistent internalised stigma which continues to hinder health-seeking behaviour.

Anticipated stigma from communities, friends and health workers was commonly described during the formative phase, seemingly decreasing over time. There were only a couple of accounts at baseline and endline, linked to persistent fears of physical violence from health workers, which was driven by missinformatione.g., that limbs would be amputated without warning. Anticipated stigma was detrimental to formal health-seeking behaviour and commonly attributed to reintegration fears. Violence perpetrated by persons affected was rare, with a few accounts at formative and baseline and just one respondent highlighting it at endline, reportedly learning to control his temper through the REDRESS intervention.

### How do gender and other social dimensions shape experiences of violence?

Gender influenced experiences of violence in numerous ways, including the prevalence, manifestation and perceived determinants. Experiences often mirrored gender norms and intersected with other forms of social dimensions, including socioeconomic status, education, family status and reproductive roles.

At the societal level, only women and girls described dropping out of school due to stigma, and whilst the prevalence of violence from formal health workers was similar between genders, only women described being denied healthcare and experiencing physical violence at the hands of formal health workers. Moreover, at endline, accounts of improved relationships with health workers were predominantly from men. Regarding experiences of violence from informal providers, experiences were similar across genders, though the only reports at baseline were among women and included harmful treatment and extortion.

Whilst women did highlight emotional violence at the community level, namely at baseline, and commonly described being excluded from community meetings, enacted community violence was seemingly more prevalent among men. Men commonly reported emotional violence across the period and physical violence was more commonly described by men.

At the relationship level, accounts of violence from family and friends was relatively similar between women and men, though the only single accounts of persistent stigma from friends at baseline and endline were from men. Generally, men attributed violence to perceptions among their family and in-laws that they are unable to provide financially, illustrating the intersection between skin NTDs, family status and gender norms.

In terms of IPV, accounts of abandonment were marginally more prevalent among women, with half of the respondents describing being abandoned by their husbands or boyfriends during the formative phase, including being repeatedly “dashed” by multiple partners over the course of their illness. This was largely attributed to women becoming pregnant or flare ups in their condition post-birth, with women commonly recalling being impregnated and abandoned by multiple men. Typically, men promised to care for them and their children financially, before invariably abandoning them and withdrawing financial support, driving women further into vulnerability. A couple of other women also highlighted feeling unable to leave their partners, due to socioeconomic dependence. This highlights the intersection between reproductive roles, post-partum vulnerability and poverty, which necessitate “patriarchal bargaining” for socioeconomic security [50]. Other manifestations of IPV included a young girl being coerced into sex work by her landlord and receiving death threats from her father after becoming pregnant, as well as a young woman forced into an arranged marriage with an older man, resulting in the termination of her education. At baseline, one woman also recalled being taunted by her husband after she urinated in their bed, due to her condition.

*Because if this foot business any man that approach her she can agree but soon the man take child from her than the man will just dash her. That thing it worrying her. Because anybody come he say oh I will do the other one, soon now they take pregnancy, hmmm be running leave alone the children with her. So, that thing is worrying her too (Female affected by LF, Maryland, formative phase)*

Conversely, several men described being abandoned at formative due to their perceived socioeconomic failures, as well as impotency linked to violence in one case, with some men affected by LF also reporting difficulties finding partners. Typically, the community and parents played a role in encouraging women to leave their husbands or partners affected by skin NTDs, due to perceptions of them being unable to contribute at the household and community level, illustrating the intersection with entrenched norms of masculinity and structural economic vulnerability. However, a couple of men recalled re-marrying after being abandoned, with one reportedly committing adultery during his second marriage – diverging from the experiences of women. There were no accounts of SGBV at baseline and endline.

*Yeah, the woman left me because nothing I can do, to put food in the house see it’s hard, no money, so all of that when you look at it, she say I can’t be suffering under this man with this kind of sore, people telling her all type of talk, pah what are you doing with this kind of man? So that what make she left. (Male affected by BU, Bong, ILN, formative phase)*

Among persons affected who described abandonment, women were generally aged 26–49 or younger, whilst men were typically aged 49 or older, Illustrating the unique ways that age and gender intersect for men and women. Women’s risk of abandonment was highest during their reproductive peak, whilst the abandonment likely intersected with economic decline among men.

Whilst both women and men highlighted associated stigma, wives and mothers were reportedly most impacted, including some accounts of community-level physical violence due to women’s persistent support of their children or husbands affected by skin NTDs. Moreover, wives, sisters and mothers were typically responsible for caregiving, such drawing water, bathing and cooking for persons affected, with only a few accounts of supportive husbands or sons. This included managing the socioeconomic burden associated with treatment costs and loss of household income, through contributing to farm work or taking up additional employment. In some cases, the absence of support from other family members necessitated children taking up begging or helping on farms, likely impeding educational attainment. These accounts highlight how gendered caregiving roles, age and collective norms converge to produce a gendered triple burden, wherein women and girls absorb the loss of income and increasing unpaid care responsibilities, in addition to compounding associated stigma

*Before I came here my family and community dwellers started hitting me already. They went as far as driving me away saying my children and I should go in the bush. But my mom refused and they started to hit her too. But this man here [pointed to the person] went in the village and brought my children and I here. Since I came my relatives don’t visit me at all] (Female affected by leprosy, Ganta rehab, ILN, formative phase)*

However, some wives of men affected described being embraced and appreciated by the community for their roles caring for men affected who were blind. Although the prevalence and manifestation of internalised stigma was common between genders and largely owed to physical condition, men consistently described shame linked to socioeconomic failures and feeling emasculated. Men also reported internalising witchcraft allegations more often and emphasised the negative mental health impacts more commonly than women. Conversely, women linked internalised stigma to their inability to contribute to household tasks, wear school skirts and lie with their children. Whilst anticipated stigma was more common among women at the formative stage, the few accounts at baseline were among men. All accounts of violence perpetrated by persons affected were narrated by men, except for one alleged incident where a woman bit her husband, described by an informant.

### How does disease condition shape experiences of violence?

Overall, out of all disease conditions, people affected by leprosy most frequently described having experienced stigma and violence. Persons affected commonly described physical and emotional violence at the community level, stigma from friends, family, IPV and associated and anticipated stigma, at the individual level, due to witchcraft beliefs and transmission fears. Participants consistently emphasised the weight of the term ‘leprosy’ due to its historical association with moral impurity, poverty, societal segregation and its depiction in the Bible, commonly highlighting the visceral fear which spread through communities when they learnt of their diagnosis. This resulted in the dehumanisation and systematic exclusion of persons affected, who accounted for some of the most disturbing accounts of physical violence and community exile, as well being restricted of basic resources e.g., communal waterpoints and housing, illustrating structural violence Consequently, suicidal ideation was most common among persons affected by leprosy, followed by BU.

However, participants commonly described the urgency with which care was sought, potentially illustrating how the historical weight of leprosy legitimises care. Whilst there was a narrative of health workers turning leprosy patients away following the war, experiences of violence in the healthcare setting were less common, with some people highlighting positive experiences. Participants spoke fondly of their time at Ganta rehabilitation, where there was a sense of “love” and understanding among persons affected, staff and the wider community. Whilst this acted as a protective factor, persons commonly highlighted reintegration fears, perpetuated by families abandoning them upon admitting them for care.

*it got a big name. sometime when they called you leprosy you know even way back those people in the Bible hmm, the name is big, is fearful. The name look fearful. When they called you leprosy patient sometime people, people that are … people that don’t know they will sometime feel that you … in fact they will come to … not to even be a human among them. they come to you different (Male affected by leprosy, Lofa, IDI, baseline phase)*

Stigma from friends, community-level emotional violence and anticipated stigma was more commonly described among people affected by BU, especially women. Some persons affected highlighted positive relationships with formal health workers and informal providers. Notably, school dropouts were most common among people affected by BU and LF. The latter was also associated with physical violence from communities, though people affected by LF also described supportive families and communities. This included a couple of women married to men affected by BU and LF who were blind and emphasised their community’s appreciation and support for their caring roles, reflecting the hierarchies of stigma in relation to more biomedically recognisable and ‘acceptable’ forms of disability, which are not associated with harmful beliefs.

Finally, some of the most distressing accounts of physical and emotional violence were recounted by a married couple, both affected by leprosy. This included the couples rightful land and babies being confiscated from them by their own families, and refusal to let their children visit due to fear that the children would be infected. This demonstrateshow a lack of critical knowledge and understanding about causation relating to limited education, combined with historical fear of leprosy relating to biblical beliefs and a lack of confidence in the health system’s ability to manage the condition, shape the discriminatory attitudes and practices of family members, illustrating compounding, intersectional stigma.

*When I went to the colony there, my thinking was worrying me about the children. Because, the children, I supposed to be with them, then they take me from them, they carry me and people will not allow for the children to come to where I am. Because I am sick and they say maybe when you carry the children there, maybe they will get sick oh. So the children were with the man to where I came from. It was part of my worriness (worries). But … once I was taking medicine so I asked. I say y’all (you people) can’t bring the children they say “No! Take your medicine. Don’t worry!” So that’s what used to happen to me. (Female affected by leprosy, Wuo Town (ex-leprosy colony) Nimba, IDI, formative phase)*

## Discussion

Stigma and violence are key social determinants of health which threaten progress towards the WHO 2030 roadmap and universal health coverage, if left untreated. This study sought to explore experiences of stigma and violence among persons affected by skin NTDs in Liberia over the course of the REDRESS intervention from 2019 to 2023. REDRESS aimed to co-develop, pilot and evaluate a person-centred, integrated skin NTD programme. Our study suggests that the intervention period was associated with a shift in community and health worker attitudes and beliefs, reducing descriptions of some forms of stigma and violence and ultimately strengthening participation of persons affected in health systems and society. However, internalised stigma and emotional violence, commonly attributed to myths and misconceptions, remain prevalent. Gender dynamics and supernatural beliefs play a critical role in driving discriminatory beliefs which contribute to experiences of stigma and violence, as well as constraining coping strategies, compounding vulnerability.People affected by leprosy most frequently describe stigma and violence, as compared with people affected by other skin NTDs, though living in Ganta and surrounding communities was considered protective, due to discriminatory views being less prevalent.

### Conceptual framing of the relationship between skin-NTDs, stigma and violence

**Fig. 4:**
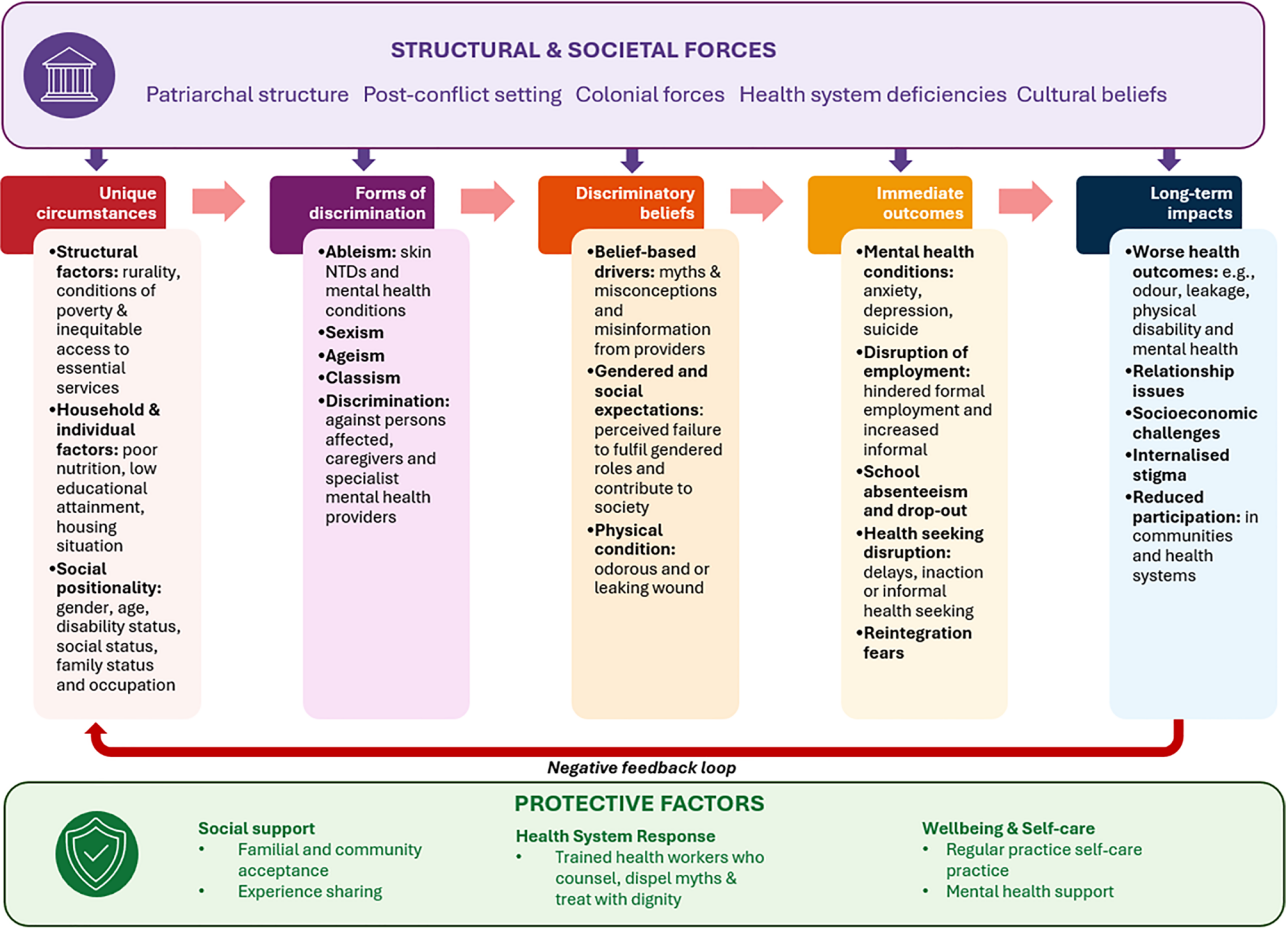
Conceptual framing of the relationship between skin NTDs, stigma and violence. Certain unique circumstances, including conditions of poverty, inequitable access to essential services and mental illness increase the risk of contracting skin NTDs. Wider forms of discrimination, including sexism and ableism drive discriminatory beliefs related to myths and misinformation, gendered and social expectations and physical condition, resulting in experiences of stigma and violence. Immediate outcomes include mental health conditions, disruptions to employment and education, as well negative changes in health seeking behaviour and reintegration fears. In the long-term, these impacts drive worse health outcomes, relationship issues, socioeconomic challenges, internalised stigma and reduced participation in communities and health systems. There is a negative feedback loop, wherein outcomes reinforce poverty and vulnerability, perpetuating skin NTDs, stigma and violence. Intersecting structural and contextual factors reinforce vulnerability to stigma and violence across all levels. Key protective factors include social support, health system responses and wellbeing & self-care

Our study demonstrates that the legacy of abuse is far reaching and complex, imparting multifaceted health outcomes which are driving intersecting inequities across numerous levels. Based on our findings and existing literature [3, 51, 52], we present a conceptual framing (Fig. 4). We explore how intersecting inequalities are shaping relative power and experiences of violence and reflect on the implication of findings in relation to the literature, before presenting evidence-based recommendations for addressing stigma and violence in persons affected by skin-NTDs.

### Drivers of inequality, contributing to the experience of stigma and violence

Experiences of stigma and violence, including the vulnerability, manifestation, severity and capacity to cope, are shaped by intersecting axis of inequality which operate across multiple levels, compounding vulnerability. As discussed, wider structural and societal forces, including health system deficiencies, cultural beliefs and attitudes towards skin NTDs and mental health and Liberia’s post-conflict setting, compound discrimination and disempowerment through cumulative, cross level effects (Fig. 4).

People’s personal social dimensions shape their vulnerability to contracting skin NTDs. Rural settings are frequently associated with poverty, gender inequality, inequitable service access and harmful practices, increasing the risk of skin NTDs [5]. Risk is also impacted by social dimensions (e.g., low educational attainment and inadequate housing) [53, 54], as well asexisting mental illness conditions [3].

These societal dimensions interact with wider forms of discrimination, including ableism and sexism, to perpetuate harmful discriminatory beliefs. Important discriminatory beliefs highlighted in the study related to spiritual beliefs and gender norms and expectations, which intersected with other social dimensions, such as poverty, age, education and health system deficiency to shape experiences of violence and stigma – we explore these below.

One of the most striking findings was the extent to which persons affected by skin NTDs are being stripped of their identity as human beings, with numerous distressing accounts of physical violence. These accounts were primarily linked to myths and misconceptions, embedded in spiritual beliefs. This aligns with the literature, which attributes violence to perceptions that people with disabilities, including skin NTDs, are demonic, non-human entities, or that they are cursed or witches, or that their experience is a punishment from God [3, 33, 55]. A systematic review described people affected by leprosy being shunned from communities and forced to live in the bush, where they inevitably die, due to leprosy being ascribed to ‘sinfulness’, or that it ‘runs in the family’ and is self-inflicted [5, 56–58]. Similar accounts were reported in Ghana and Nepal, where physical violence and exclusion from communities was common among men with more severe deformities, echoing our findings [33, 56, 58].

One of the most dehumanising accounts was experienced by a married couple both affected by leprosy, who were stripped of their rightful land and their own children, illustrating compounding, intersectional disempowerment across multiple structural and interpersonal levels [43]. These harmful misconceptions are deeply rooted in society; leprosy, widely considered an emblem of health-related stigma [3], dates to 1550 BC [59] and was cited as a “metaphor for sin” and punishment from God in parts of the Old Testament in the Bible [60, 61]. Leprosy-related stigma is also ascribed to witchcraft misconceptions, transmission fears and its association with “inferior people” due to colonialism and historical public health interventions centred around segregation in leper colonies, perpetuating fear, illustrating the cumulative and reinforcing effects of structural violence [61–66]. Interestingly, we also found that men who had skin NTDs but were blind, were treated with respect and their wives were appreciated by the community, suggesting some disabilities, including those that present biomedically and are not infectious, are considered more socially acceptable [67]. It also speaks to research which demonstrates that the incidence of violence declines with the increasing disease severity, as communities and families are less likely to misattribute inadequate participation/contribution to laziness – individuals who are blind, are considered exempt from social norms surrounding contribution to society [55, 67].

Given the deep-rooted beliefs in supernatural causes in Liberia, it is unsurprising that traditional medicine forms a key component of health care for many Liberians – and hence, the REDRESS intervention. It is worth noting that whilst REDRESS included intervention components aimed at improving the relations between health workers and informal providers, their relationship has historically been contentious, due to conflicting beliefs and competition for service revenue [68]. This may have potentially influenced the results, inflating narratives of violence and stigma reported about formal health workers from informal providers and vice versa. Throughout the study, participants consistently emphasised the degree of community influence and trust experienced by TH/FHs, who were considered more effective and approachable [68]. Whilst some described declines in violence from informal providers, many emphasised ongoing challenges with the actors perpetuating myths and community stigma and some informal providers continuing to express discriminatory views towards people affected by skin NTDs and mental health conditions at endline. Although the prominent role of traditional medicine in treating skin NTDs in Africa been highlighted in the literature [34, 68–72], there is a lack of evidence around the role of TH/FHs in perpetuating these myths and misconceptions.

We found that gender intersects with other forms of advantage and disadvantage and identify factors, such as age, to shape experiences of stigma and violence, supporting the literature [5, 12, 30, 33, 34, 67]. For instance, most school dropouts were among girls and violence was linked to perceived failure to fulfil gender roles, echoing evidence and illustrating the intersection between gender, age and educational attainment [5, 7, 33, 73]. Accounts of abandonment and adultery were marginally more common among women and were typically attributed to becoming pregnant or disease flare ups after giving birth. This illustrates the relationship between vulnerability, reproductive capacity and age, mirroring findings from Alderton et al. [33] and other studies [30, 74, 75]. the literature suggests women and girls bear responsibility for unpaid caring responsibilities, hindering education and employment [33, 74, 76, 77]. Women were also more commonly denied healthcare and experienced physical violence from health workers, with improved relationships with health workers more prominent among men at endline. This speaks to the health inequities which women with skin NTDs face, shaped by patriarchal structures and institutionalised discrimination within health systems, manifesting as structural violence [33, 78–80]. Additionally, women more commonly reported exclusion at the community level, from meetings and events, mirroring findings in Ethiopia [5, 81], Tunisia [82] and Nepal [83] which illustrate lower social participation among women. These impacts likely contribute to the statistically higher levels of internalised stigma and mental health issues among women compared to men, reported elsewhere [30, 37].

Our study found that men’s inability to provide financially and dependence on women for livelihood activities was a key determinant of internalised and enacted stigma across all levels [7]. Interestingly, spousal abandonment was only marginally more common in women, conflicting with a global systematic review, predominantly in the African region, which asserts women are more vulnerable to abandonment than men [33]. Moreover, witchcraft allegations and the internalisation of beliefs were generally more commonly described among men, diverging from the literature in Central and West Africa, which asserts that women and children are more likely to be accused of witchcraft [55, 67]. Additionally, the few accounts of challenges finding partners were among men, deviating from the evidence base, which asserts women are more susceptible, partially due to notions of men being household earners [5, 84–89]. Whilst the accounts of violence perpetrated by persons affected were rare, the few narratives were from men; this speaks to evidence from Zimbabwe and Ethiopia, where notions of masculinity, dominance and the “biblical concept of submission” perpetuate violence as a means of obtaining power by persons affected by skin-NTDs, including against women with disabilities – though this was not captured in our study [5, 90].

The fact that there was only one personal account of SGBV, reported by a woman at baseline, diverges from the literature and may be due to cultural beliefs that incidences of violence such as SGBV be dealt with privately, perpetuated by gendered power dynamics [78]. Moreover, REDRESS did not explicitly explore SGBV – a notoriously sensitive and difficult subject to research, since this was not the direct focus of the study. Nevertheless, our study clearly illustrates how gender shapes experiences of violence, influencing health-seeking behaviour and ultimately, gender inequality. It is also probable that women less commonly described other forms of violence, due to their unequal stance in society. Despite this, as the systematic review from Alderton et al. [33] emphasises, there is limited research into the gendered experiences of skin NTDs.

### Multifaceted and intersectional outcomes

Violence and stigma commonly result in persons affected, primarily young women, dropping out of school. This is hindering women’s socioeconomic independence, and may increase dependence on men, possibly increasing vulnerability to abusive partners, as highlighted by some women in our study [5, 33, 91]. Dropping out of education also jeopardises opportunities for peer relationships and societal acceptance, driving generational poverty and internalised stigma [5, 55, 92]. This, combined with the burden of unpaid caregiving, contributed to most women affected by IPV in the Alderton et al. [33] review being uneducated and unemployed.

Persons affected also described challenges with employment, causing some to adopt informal opportunities, including sex work and begging – this, combined with families and communities halting support and even confiscating farming land in one case, illustrates the complex livelihood challenges associated with skin NTD stigma [35, 93]. Our findings align with Alderton et al. [33], wherein 63% of studies mentioned job loss, especially among men. Aside from reinforcing socioeconomic inequalities and poverty, reported in 48% of the studies in the review, high unemployment is driving negative stereotypes of persons affected failing to contribute to society [33, 94].

Unemployment is also associated with mental health issues [3, 33]. Persons affected and informants consistently emphasised the detrimental impacts of stigma and violence on mental health, with numerous accounts of depression, anxiety, suicidal ideation and one reported case of suicide, supporting the literature, wherein Alderton reports that 41% of studies reported suicidal ideation related to skin NTDs [5, 73, 95]. Devries et al. [96] also emphasises that mental illness exacerbates violence risk, through mental health related stigma, further driving self-isolation [5, 73]. Moreover, persons affected by violence typically have limited “resources for change” (e.g., financial resources)– this, combined with restrictions to bathing materials and water sources reported by respondents, may be impeding disease self-management [5]. Additionally, the direct physical impacts of experiencing violence may worsen physical disability [97] and may have sexual and reproductive health impacts, as mentioned by one man who became impotent [94]. Addressing the psychosocial impacts of skin NTDs is essential. The REDRESS intervention has demonstrated the value of integrating mental health support within NTD programs, and these findings highlight the importance of sustaining the integration of mental health services within NTD programmes.

### Long-term impacts of experiencing stigma and violence

Finally, persons affected, and informants consistently emphasised how experiences of stigma and violence are impeding health-seeking behaviour, echoing the evidence base [3, 33].

Whilst the nature of this paper does not enable us to quantify the prevalence of stigma and violence (see McCollum et al. [30] for a quantification at the baseline REDRESS phase), the findings do suggest that REDRESS had positive impacts with reports of decreased internalised stigma, primarily among PSG members. However, our findings suggest that emotional violence and internalised stigma remain prevalent. This aligns with the literature, wherein numerous studies, including systematic reviews, demonstrate that internalised stigma, characterised by shame and low-self-esteem is highly prevalent among persons affected by skin NTDs continuing interventions [3, 33, 35, 37].

Literature emphasises the detrimental impacts of internalised stigma on health-seeking behaviour, mental health and participation, forming a vicious cycle wherein persons affected isolate themselves, further driving community stigma and poor health outcomes [3, 98, 99]. This, combined with the fact that internalised stigma entails “the active absorption and assimilation of negative societal attitudes by individuals about themselves” which are mirrored in society, makes internalised stigma particularly difficult to address [73]. Similarly, the literature demonstrates that emotional violence is common among persons affected by skin NTDs, potentially as it is considered a more socially acceptable form of violence which can be perpetrated more casually, with limited repercussions [67]. These findings are particularly concerning as internalised stigma is associated with hindered health-seeking behaviour, reintegration and suicide [3, 73, 100, 101].

Internalised stigma, including the belief that skin NTDs are incurable or caused by supernatural forces, as well as community stigma, resulted in delays or inaction, supporting the literature [3, 30, 92, 94, 102, 103]. Moreover, some people affected by leprosy highlighted reintegration fears, with some reportedly wishing to stay in colonies where they felt accepted, due to internalised stigma [101]. This is particularly concerning given that these were the two most persistent forms of violence at endline, supported by Koschorke et al. [3] whose review found that internalised stigma was as prevalent and impactful as enacted stigma. Enacted and anticipated violence from health workers and informal providers, including the perpetuation of myths and misconceptions, was also prevalent and hindered health-seeking behaviour, supporting the literature [3]. Notably, some women were reportedly denied health care, illustrating the intersections with gender and patriarchal structures.

Overall, our study elucidates a vicious, mutually reinforcing and intersectional cycle between poverty, skin NTDs and stigma and violence (Fig. 4) [3]. People affected living in poor, rural areas, with limited access to education, essential services and adequate housing and existing disability (including mental health conditions) are more likely to contract skin NTDs., Wider forms of discrimination, including sexism and abelism drive stigma and violence at numerous levels, resulting in unemployment, low educational attainment, health seeking disruption and relationship issues, with women and girls disproportionately affected. This experience results in numerous barriers to participation and inclusion, including socioeconomic challenges, mental health conditions, inadequate support, discriminatory attitudes and practices from health providers and internalised stigma, shaped by internalising beliefs about skin NTDs being self-inflicted, incurable, caused by witchcraft or curses and of themselves being burdens or non-humans. This impedes health-seeking behaviour (i.e. Delays, inaction or alternative health seeking), limiting health outcomes (including odours and leakages which are highly stigmatised) and exacerbating stigma across all levels, including internalised stigma, which drives further withdrawal from society. This includes either consensual or forced exile from communities and families anddrives generational poverty, perpetuating notions of persons affected being burdens who fail to contribute and lack humanity. Ultimately, this decreases acceptance, justifying and perpetuating social discrimination and isolation. Therefore, addressing stigma and violence towards persons affected by skin NTDs is essential on numerous levels, as it will support progress towards numerous SDGs, including the universal health coverage and gender equality, as well as the WHO 2030 roadmap. To reach the last mile, an integrated, person-centred approach is crucial.

### Evidence-based recommendations for addressing stigma and violence

Considering the study findings and our conceptual framework, we propose the following recommendations for addressing stigma and violence among persons affected by skin NTDs.

It is widely acknowledged that there is limited evidence on integrated interventions to improve the wellbeing of persons affected by skin-NTDs and implementation research has a central role to play in designing and implementing effective interventions and disentangling the relationship between stigma and skin NTDs, especially in relation to social determinants [3]. The intervention showcases the value using participatory action research to co-design sustainable, community-led interventions to strengthen person-centred health systems and improve mental wellbeing. As the intervention was co-designed and implemented with the Liberian MoH and is in keeping with the ECP [38], it is being adopted in national policy through the Liberia NTD Masterplan (2023–2027). Therefore, we recommend that future implementation studies adopt similar participatory approaches to ensure meaningful participation and engagement of persons affected and ensure that interventions are contextually relevant and sustained.

To address persistent forms (emotional and internalised) of stigma and violence, we need to address myths, garner community support, provide mental health support and shift the self-perception of persons affected. Such interventions must be sustained, multi-pronged and engage people from across the health system and beyond, to be effective in overcoming existing norms, social and structural factors [3, 73, 99, 104]. WHO [13] highlight the role of CBGs (e.g., PSGs) in achieving this, as they present a sustainable, person-centred approach to addressing stigma and improving health outcomes, through engaging in self-care, mental health, livelihoods and advocacy [36, 105]. CBGs are typically governed by persons affected, supporting genuine participation and self-efficacy [106–109]. This, combined with experience sharing, livelihood and community development activities (e.g., fixing community infrastructure) delivered by members, promote community acceptance and positive self-perceptions and empowerment [110, 36, 106, 111]. Therefore, we recommend that future skin NTD interventions incorporate CBGs.

Given the central role of informal providers in shaping myths and misinformation and the lack of available literature, future skin NTD interventions should actively engage informal providers, to utilise their role in active case finding and addressing stigma and myths and misconceptions. Fully considering and addressing local beliefs is essential [3]. To effectively harness informal providers, more evidence is needed.

Our study shed light on the role of gender dynamics in shaping the vulnerability, experience and resilience to stigma and violence, addressing evidence gaps highlighted by Koschorke et al. [3]. Further research into the gendered dimensions of stigma and violence in needed, as is the collection of gender disaggregated data and the active engagement of women throughout the research cycle. We also urge future research projects to consider the unintended consequences of gender hierarchies on findings (e.g., separating FGDs by gender and potentially age groups) and to approach the experiences of violence and stigma with caution and sensitivity, including establishing referral mechanisms prior to data collection. Strengthening the evidence base will enable gender transformative interventions to address stigma and violence experienced by people affected by skin NTDs. Additionally, health systems should integrate SGBV prevention and responses [79].

### Strengths and limitations

This study makes a novel contribution to the limited evidence on experiences of stigma and violence among persons affected by skin NTDs in Liberia. We used longitudinal data and triangulated different participatory methodologies and types of respondents. This, combined with the active role of Liberian co-researchers (including person affected) and research fellows in data collection and analysis, supports the credibility of findings. However, there are some limitations to consider; firstly, the tools used throughout the study period were not uniform and did not always focus or probe on stigma and violence. Furthermore, as mentioned in the discussion, some types of violence, such as SGBV are much more stigmatised than others, due to social norms. Moreover, REDRESS did not focus on SGBV, with a more general focus on stigma. The implication of this is that we cannot quantify the incidence of violence and stigma, and readers should be cautious when interpreting the results. We know that gender and disease condition intersect with other identity factors, such as age and geographical location to shape experiences of stigma and violence. Unfortunately, the nature of the available data restricted the extent to which these dimensions could be explored. Therefore, we suggest that future studies explore these additional factors. Additionally, it is important to note that there are longstanding tensions between formal health workers and informal providers, which came through strongly throughout REDRESS dataset. Whilst the intervention did strengthen the relationship and trust between these actors, a continued lack of trust between these groups may introduce bias and hinder interpretation of results. For instance, the concern surrounding continued violence from informal providers which came through strongly in conversations with formal health workers, may reflect their negative relationships with informal providers. Finally, whilst much of the data collection and analysis was led by Liberian colleagues, including those with lived experiences of skin NTDs, the analysis and write up of this study was conducted by the lead researcher (IH) who is a white European woman. Whilst the lead researcher has spent time in Liberia, she does not have lived experience of skin NTDs, or a deep contextual understanding of Liberian cultural practices and gender norms, which may have biased interpretation of the results. To mitigate this, interpretations and findings were discussed with Liberian colleagues, who also reviewed the transcript, and the lead researcher actively reflected on her own positionality throughout the process.

## Conclusion

Overall, the study illustrates that stigma and violence is prevalent across different levels and whilst the holistic health system strengthening intervention through REDRESS did improve attitudes and practices, internalised stigma and emotional violence persist. Societal factors, such as traditional beliefs and gender norms are intersecting with identity factors, including gender and disease condition, shaping experiences and resilience. Stigma and violence causes multifaceted health impacts; aside from impeding the equitable delivery of skin NTD programmes, stigma and violence is perpetuating mental health issues and societal inequities, risking progress towards the SDGs. More evidence is needed to support the implementation of contextually relevant person-centred interventions, and the role of the informal health sector and gender must not be ignored.

## Data Availability

Given that this study was conducted in a limited number of counties and details on the gender, condition and cadres of respondents was disclosed for analysis purposes and explores a highly sensitive subject, making transcripts readily available may risk respondents being identified. For persons affected, this may result in further experiences of stigma and violence and may negatively affect their relationships and wellbeing. Therefore, we request that this study be exempt from making data publicly available.
